# Clinical spectrum and risk factors associated with asymptomatic erosive esophagitis as determined by Los Angeles classification: A cross-sectional study

**DOI:** 10.1371/journal.pone.0192739

**Published:** 2018-02-27

**Authors:** Akhilesh Kumar Kasyap, Shiv Kumar Sah, Sitaram Chaudhary

**Affiliations:** 1 Department of Medicine, Gastroenterology unit, National Academy of Medical Science, Bir Hospital Mahabauddha, Kathmandu, Nepal; 2 Faculty of Pharmaceutical Science, Purbanchal University, Little Buddha College of Health Science, Minbhawan, Kathmandu, Nepal; University Hospital Llandough, UNITED KINGDOM

## Abstract

**Background:**

Gastro esophageal reflux disease (GERD) is a chronic and recurrent disease, and it varies in regions. However, to date, there are no reports available on clinical features and the risk factors for the asymptomatic reflux esophagitis in Nepalese adults.

**Methods:**

Data were gathered from 142 erosive patients who had undergone endoscopy at Bir Hospital, Kathmandu. Los Angeles classification was used to grade the severity of the disease. Patients were interviewed to find out the presence of various reflux symptoms.

**Results:**

Based on the Los Angeles classification, the severity of the disease assessed was; grade A 31.8% (31/142), grade B 39.4% (56/142), grade C 33.8% (48/142), and grade D 4.9% (7/142). One hundred and twenty six (88.7%) subjects had reflux symptoms. Prevalence of asymptomatic esophagitis was 16(11.3%). Age was independently linked to asymptomatic esophagitis (P<0.05), and the odd of being asymptomatic appeared lower in younger adults (P<0.05; OR: 0.118; CI: 0.014-.994).

**Conclusion:**

A low prevalence of asymptomatic reflux esophagitis (RE) was seen. Most subjects experienced mild to moderate RE. Age remained an independent factor associated with reflux esophagitis, and the odds of being asymptomatic was lower in younger age.

## Introduction

Gastro esophageal reflux disease (GERD) is a chronic and recurrent disease. As suggested in earlier studies,[1,2,3,4] it was more common in West in comparison to Asian population. However, the recent studies have shown that number of GERD patients have been growing in Asian Pacific region [5]. This may be assumed to be an increased awareness among doctors and patients, and/or a true increase in the prevalence of the disease. On the other hand, as the incidence of GERD is increasing, asymptomatic erosive esophagitis (EE) is found to be increasing as well [6,7]. Reports on risk factors on asymptomatic esophagitis have been inconsistent between the studies. Some studies have reported smoking, body mass index (BMI) and gender as predictors of asymptomatic erosive esophagitis (EE) [8,9]. While, another study has found non- smoking and lower BMI to be significant risk factors.

Endoscopy is a useful technique for evaluating the severity of reflux esophagitis objectively [10,11]. In fact, typical reflux symptoms may not exactly correlate the presence and severity of esophagitis in an individual patient [12]. The natural course of reflux has still been a matter of debate and importantly the disease progresses over time with its changing spectrum. There are mounting evidences on clinical pictures of reflux and their associated risk factors in resources rich setting nations [6,7,13,14]. However, such studies based on endoscopy in Nepalese setting are unknown. Therefore, the present study is sought to evaluate the clinical spectrum of the esophagitis, and further to identify the predictors influencing the symptoms.

### Patients and methods

**T**his cross-sectional study was conducted at National Academy of Medical Science (NAMS), Bir Hospital, Nepal. The patients visited medical ward for physical check up and those who underwent for endoscopy at hospital, and who had follow-up endoscopy were eligible for the study. Subjects with an ulcer scar on endoscopic findings were included. Patients with a history of gastrointestinal surgery, those having taken the proton pump inhibitor, histamine-2 receptor antagonists, sucralfate, anticholinergics, or other gastrointestinal (GI) medications and non-steroidal anti-inflammatory drugs were excluded from the study.

All subjects completed a questionnaire documenting symptoms regarding esophagitis and socio- demographic status (e.g. age, sex, income and education). Patient’s personal as well as family history, lifestyle behavior, recent smoking and alcohol histories were identified by interviewing. The questionnaire was comprised of 7 questions on GERD symptoms (heartburn, acid regurgitation, dysphagia, globus sensation in the throat, hoarseness, cough, and chest pain). Each participant underwent weight and height measurements, using a calibrated scale. The body mass index (BMI) was calculated as weight (in kilograms) divided by height squared (in square meters).

### Symptoms evaluation

Patients were interviewed to find out the presence and duration of various reflux symptoms such as heartburn, dyspepsia, epigastralgia, belching and non-cardiac chest pain. Subjects who experienced heartburn or acid regurgitation one or more times in a week during the last 3 months were defined as exhibiting typical symptoms; subjects who experienced dysphagia, globus sensation in the throat, hoarseness, cough, and chest pain once or more in a week during the last 3 months were defined as having extra-esophageal reflux symptoms. Asymptomatic EE is defined as the presence of esophageal mucosal injury that is typical of GERD during upper gastrointestinal endoscopy in individuals who lack typical or atypical extra-esophageal manifestations of GERD[15].

All endoscopic examination was performed by endoscopist and was evaluated esophageal mucosal break according to Los Angeles classification ofesophagitis [11].

#### Statistical analysis

Numerical data were expressed as mean ± S.D. Frequency table was calculated and was presented in percentage (%). We performed Pearson’s Chi square test to determine whether observed difference in proportion of symptoms in study group was statistically significant. Bivariate model was used to estimate odd ratio (OR) and to determine the various predictors contributing to symptoms associated with esophagitis. A p value less than 0.05 was considered to be statistically significant. All analysis was performed using SPSS version 16.

#### Ethical consideration

The study was approved by the institutional review board (IRB) of National Academy of Medical Science (NAMS), Bir Hospital, Kathmandu. All the participants who participated in the current study were aware about the study protocol, and the subjects willing to participate in the study were written consented prior to data collection.

## Results

Demographics of the population are illustrated in Table 1. Of 142 erosive subjects, the mean ages were 45.40 ± 14.74 years. Proportion of the male patients was 74 (58.3%). Mean Body mass index was 26.23±5.24. Fifty eight (40.8%) subjects had smoking history, while 47 (33.1%) patients were alcoholics. Asymptomatic esophagitis was seen in 16 (11.3%). Severity grade with Los Angeles classification was grade A: 31 (31.8%), grade B: 56 (39.4%), grade C: 48 (33.8%), and grade D:7 (4.9%).

**Table 1:** Demographics and Patient’s characteristics.

Parameter	inference
No. of patients, n	142
Age, mean± SD (years)	46.13± 1.48
Male, n (%)	87 (61.3)
Female, n (%)	55 (38.7)
BMI, mean ± SD	26.18 ± 6.01
Smoker, n (%)	58 (40.8)
Alcohol, n (%)	47 (33.1)
Symptoms present, n (%)	126 (88.7)
Symptoms absent, n (%)	16 (11.3)
Hiatus hernia, n (%)	46 (32.4)
Peptic ulcer, n (%)	2 (1.40)
Los Angeles classification, (%)	
1. A	31 (31.8)
2. B	56 (39.4)
3. C	48 (33.8)
4. D	7 (4.9)

Characteristics of symptomatic and asymptomatic groups are illustrated in the Table 2. Sixteen subjects had asymptomatic esophagitis, with 8 comprised in the age group of 18–39 years and 8 in 40–60 years. Tendency of association towards the symptoms was significantly associated with age strata (P<0.05).

**Table 2:** Characteristics of symptomatic and asymptomatic groups.

	Symptomatic group (126)	Asymptomatic group (16)	P value
**Age**			0.026
1. 18–39	33 (26.1%)	8 (50%)	
2. 40–60	56 (44.44%)	8 (50%)	
3. >60	37 (29.36)	0	
**Sex, n(%)**			0.914
1. Male	77(61.11)	10 (62.5)	
2. Female	49 (38.88)	6 (37.5)	
**BMI, n(%)**			0.755
1. 20–24.9	50 (39.68)	7 (43.75)	
2. ≥25	76(60.31)	9 (56.25)	
Smoking (+)	52 (41.26)	6 (37.5)	0.773
**Alcoholic (+)**	44 (34.92)	3 (18.75)	0.195
**Hernia (+)**	41 (32.53)	5 (31.25)	0.917
**Endoscopic**			0.614
1. Grade A	29(23)	2 (12.5)	
2. Grade B	47 (37.30)	5 (31.25)	
3. Grade C	44 (34.92)	8 (50)	
4. Grade D	6 (4.7)	1 (6.25)	

Of 142 subjects, 10 (62.5%) male subjects had asymptomatic esophagitis. Among asymptomatic group, 8 (50%) had grade C esophagitis, 5 (31.25%) grade B, 2 (12.5%) grade A and 1 (6.25%) had grade D. Test for significance of symptoms confirmed the absence of any tendency towards an association with gender, BMI, smoking history, alcoholic history, hernia, and Los Angeles grade (P>0.05)(Table 2).

Table 3 depicts the independent predictors for asymptomatic esophagitis. Logistic regression was modeled to identify the factors associated with asymptomatic esophagitis. Age, sex, BMI, smoking, alcoholics, hernia and Los Angel grade were taken into account for the model. Age was independently linked to asymptomatic esophagitis, and the odd of being asymptomatic appeared lower in younger adults (P = 0.049: odd: 0.118: CI: 0.014–0.994).

**Table 3:** Bivariate logistic regression analysis on independent risk factors for asymptomatic esophagitis.

	Estimated value	P value	Odd	(95% CI) Lower-upper
**Age**				
18–39	-2.13	0.049	0.118	0.014-.994
**Sex**				
1. male	-1.05	0.817	1.11	0.455–2.71
2. female	-	-	-	-
**BMI**				
1. 20–24.9	0.167	0.755	1.18	0.414–3.64
2. ≥25	-	-	-	-
Smoking (+)	0.292	0.516	1.34	0.554–3.34
Alcoholics(+)	-0.25	0.613	0.77	0.287–2.08
Hernia (+)	-0.77	0.873	0.926	0.359–2.38
**Endoscopic**				
1. Grade A	0.229	0.845	1.25	0.126–12.53
2. Grade B	0.135	0.906	1.14	0.121–10.61
3. Grade C	0.675	0.548	1.96	0.217–17.74
4. Grade D	-	-	-	-

## Discussion

We reported the clinical characteristics and the symptoms presents in esophagitis patients. In this study, we applied Los Angeles classification of esophagitis to evaluate the severity of disease [11]. According to the Los Angel classification grade, most of the patients had a diagnosis of grade B (39.4%), followed by grade C (33.8%), grade A (31.8%) and grade D (4.9%). Majority of the reflux esophagitis was mild to moderate in our study and was consistent with the other oriental countries [14,16,17].

In our study, gastroesophageal reflux symptoms, which are either typical or atypical, were reported by 88.7% of patients with endoscopy proven reflux esophagitis, and 11.3% patients were asymptomatic. The prevalence of asymptomatic esophagitis in our study population appeared to be lower in comparison with the previous study explored by Jae Heecho (45.3%) and Sung Hoon Jung (36.7%) [18,19]. However, our finding was consistent with the pervious findings explored by Wang FW (12%), and Nagahara A (11.6%) [9,20]. In previous studies, the prevalence of endoscopy-proven reflux esophagitis was 11.8%-27.4%, and 37% of EE had typical symptoms [14,21,22]. A study from Korea reported that the prevalence of RE was 6%, with 45.3% asymptomatic [18]. These variations in symptoms may account for the factor whether extra-oesophageal symptoms were included or not. Available literature suggests that extra-esophageal symptoms are associated with GERD[23],and in our study population extra-esophageal symptoms were taken into consideration for symptomatic esophagitis.

Of 126 symptomatic patients, our study demonstrated that more than 50% had mild to moderate disease severity, and 50% had grade C. Only about 5% subjects had severe esophagitis (grade D). Test for significance between severity of the disease and symptoms present were not correlated significantly (p<0.05), which corresponds well that of the previous study [19]. This implies that severity of disease is not the predictors of symptoms manifestation, and judgment of severity based on the symptoms is not crucial.

A logistic regression was modeled to illustrate thepredictors associated with asymptomatic esophagitis. Age, sex, BMI, smoking history, alcoholic history, disease severity were included for analysis. Our study demonstrates that ages (18–39 years) were independently linked to asymptomatic esophagitis (P = 0.049: odd: 0.118: CI:0.014–0.994). Earlier study has showed that gastrointestinal disorders symptoms in elder patients may be slight or atypical, resulting in a delayed diagnosis [24]. This is hypothesized that the number of myenteric esophageal neurons at elderly decreased and effect of nerve stimulation varies accordingly [25].

Available literature suggests that male sex, hialtal hernia, smoking, alcohol and obesity are independent risk factors for aymptomatic esophagitis [13,14,18]. However, in our study, Sex, BMI, alcohol history, Hiatus hernia were not linked to asymptomatic esophagitis, and the result was in consonant with the study conducted in large population by Akihito Nagahara [20]. A possible reason for this may be the fact of the low proportion of these predictors in the asymptomatic groups in this particular population that might have affected the association.

We acknowledge that this study has been accomplished with few limitations. The major limitation of our study was being a relatively small sample size (only 16 asymptomatic subjects) which was significantly lower than the published article. Owing to small sample size, the association between asymptomatic esophagitis of different predictors might have been greatly affected.

Also, H. Pylori infection rate was not evaluated in our study, however, it is suggested that infection is associated with less and milder esophagitis cases.

## Conclusion

Our study demonstrated a low prevalence of asymptomatic RE in Nepalese adults. Most subjects had mild to moderate esophagitis as graded by Los Angles classification. Age remained an independent factor associated with reflux esophagitis, and the odd of being asymptomatic was lower in younger age.

## Abbreviations

BMI: body mass index
GERD: gastro esophageal reflux disease
NAMS: National academy of medical science
RE: reflux esophagitis
